# A Rare Talar Fracture Caused by Direct High-Impact Trauma

**DOI:** 10.3390/reports9030279

**Published:** 2026-08-21

**Authors:** Nikolaos A. Stavropoulos, Fotios Kantas, Dimitrios V. Papadopoulos, Rizos Tsaknis, Vasileios S. Nikolaou, George C. Babis

**Affiliations:** 1 Second Department of Orthopaedic Surgery, School of Medicine, National and Kapodistrian University of Athens, “Konstantopouleio” General Hospital, 14233 Athens, Greece; 2 School of Medicine, National and Kapodistrian University of Athens, 11527 Athens, Greece; 3 General Hospital of Karpenisi, 36100 Karpenisi, Greece

**Keywords:** ankle injury, lateral process fracture, talus, snowboarder’s fracture

## Abstract

Fractures of the lateral process of the talus are uncommon injuries. We present a direct force trauma-induced talus lateral process fracture sustained in a 60-year-old male. The fracture was eventually diagnosed with computed tomography (CT) imaging and managed conservatively due to the patient’s co-morbidities and fracture type. The patient achieved an AOFAS Hindfoot Score of 90 at one year and remained asymptomatic at 78 months. Through this case, we aim to underscore the variability in clinical manifestations of the talus’ lateral process fractures as well as increase physician awareness for this type of fracture.

Ankle fractures are a common encounter in clinical practice; talar involvement, however, is relatively rare, as it accounts for less than 10% of all foot fractures. Lateral process fractures of the talus represent roughly 10% of talar fractures, highlighting their clinical relevance [1]. Lateral process of the talus fractures carry important diagnostic and prognostic issues despite their low incidence [2]. They account for just 0.86% of all ankle injuries, and as they typically present as severe ankle sprains, they are often misdiagnosed [3]. Usually referred to as “snowboarder’s fractures”, lateral process fractures of the talus usually result from axial loading combined with dorsiflexion, external rotation or eversion of the ankle [3,4,5]. Missed or delayed diagnosis can potentially lead to chronic pain, subtalar arthritis, instability, and increased morbidity [2]. We present the case of a 60-year-old male with a lateral process fracture of the talus. This report aims to raise clinical suspicion for this fracture type and highlight the diagnostic challenges associated with its detection. A sixty-year-old male patient presented due to ankle injury sustained by direct force applied to his lower limb. The mechanism of injury consisted of direct crush trauma inflicted by the fall of a goat onto the patient—an uncommon livestock-related injury mechanism; no dorsiflexion and inversion of the foot was reported by the patient. It has to be mentioned that the mechanism of injury differs from what is commonly the reason for such type of fracture that is snowboarding but also from other types such as dorsiflexion and inversion of the ankle as being described by Lunebourg et al. as well [5]. Local tenderness in the lateral side of the ankle about a centimeter distal to the tip of the fibula, swelling, limited range of motion, neurovascular status intact and inability to bear weight, along with imaging investigations, including X-rays and a computed tomography (CT) scan, revealed a fracture of the lateral process of the right talus, which was identified as the main injury (Figure 1 and Figure 2). The fracture was classified as Type 2 according to the McCrory–Bladin classification of the LTPFs [6,7] and was minimal displaced. The case was discussed by the Trauma Board, and the patient was informed accordingly. Given his co-morbidities, diabetes mellitus, and fracture characteristics and morphology, all the available treatment options and the potential risks were thoroughly discussed with the patient, and he elected to undergo conservative treatment. Therefore, a non-bearing posterior splint was applied for 6 weeks, and he was followed at the outpatient clinic on a predetermined schedule. At his 6-week post-injury follow-up time point, taking into consideration the X-ray and progressive pain relief, weight bearing as tolerated was allowed until full status was achieved (Figure 3). The functional status improved over time, as reflected by the American Orthopaedic Foot and Ankle Society (AOFAS) Hindfoot Score, which was 85 at the 6-month follow-up time point, a good result, and 90 at the annual follow-up, an excellent result [1] (Figure 4). The patient was followed according to our institutional protocol. The patient remains asymptomatic at the 78-month post-injury follow-up (Figure 5).

Lateral process fractures of the talus account for less than 1% of all ankle injuries but are missed in up to 48% of initial assessments [5]. Other injuries, including but not limited to ankle sprain, lateral malleolus avulsion fracture or fractures of the 5th metatarsal, should always be considered in the differential diagnosis. Furthermore, the treating orthopaedic surgeon should suspect ipsilateral injuries. Literature review has revealed an up to 82% of the presenting fractures of the lateral malleolus are avulsion fractures that end up being operated on with at least one associated injury [8], among them subtalar impaction accounting for up to almost 40% of the cases [3]. Our patient had no other recent concomitant associated injuries, which is relatively uncommon, and underscores the wide range of clinical symptomatology. Advanced imaging, especially CT, is deemed critical in confirming the diagnosis. CT scanning provided a definitive assessment and guided the treatment plan [2]. Initial X-rays reflected the difficulty in detecting lateral process injuries with standard radiographs alone. Furthermore, disruption of the V-shaped lateral process does not appear to be highly sensitive, with studies stating sensitivities and specificities up to 77% and 59%, respectively [1]. Therefore, in cases of high clinical suspicion, CT should be considered as the next investigative step [1]. Although CT is the gold standard for fracture visualization, MRI may be useful in cases with persistent pain and tenderness, when occult osteochondral injury is suspected or ligament injuries coexist. Conservative management is generally recommended for non-displaced fractures or in cases of small or even intermediate-sized fractures [1,3,9]. Surgically treated patients do not consistently achieve better functional outcomes compared with those managed conservatively, supporting the non-operative management in the aforementioned selected cases [1,3]. Furthermore, delay in diagnosis and co-morbidities have been identified as factors that influence the overall management in terms of conservative versus operative management [1]. Sariali et al. identified and published the high risk of osteoarthritic incidence in the cases of delayed diagnosis compared to early diagnosis [10]. Our patient achieved excellent long-term functional outcomes as depicted by American Orthopaedic Foot and Ankle Society (AOFAS) Hindfoot Score, having undergone conservative treatment [1,11]. The patient achieved an excellent score of 90, a result reported in only 17% of surgically treated patients, supporting the success of conservative management in this case [1]. The patient remains asymptomatic 78 months post-injury, which contrasts with the higher complication rates reported in surgical cohorts. In an average follow-up of 44 months, up to 50% of surgically treated patients develop post-traumatic symptoms such as subtalar arthritis, emphasizing the potential benefits of conservative management in non-displaced fractures [6]. Possible limitations could be the fact that no other scores like the FAAM, the PROMIS, VAS pain or the SF-12 were used and the lack of post-injury computed tomography (CT). We aim to emphasize the importance of maintaining a high index of clinical suspicion for lateral process fractures of the talus, especially in patients presenting with lateral ankle pain and swelling. Utilization of CT imaging and the high prevalence of ipsilateral injuries are of paramount importance. Timely management and a thorough clinical examination and evaluation, even in cases of delayed diagnosis, are crucial to optimize long-term functional outcomes and avoid common post-traumatic symptoms.

## Figures and Tables

**Figure 1:**
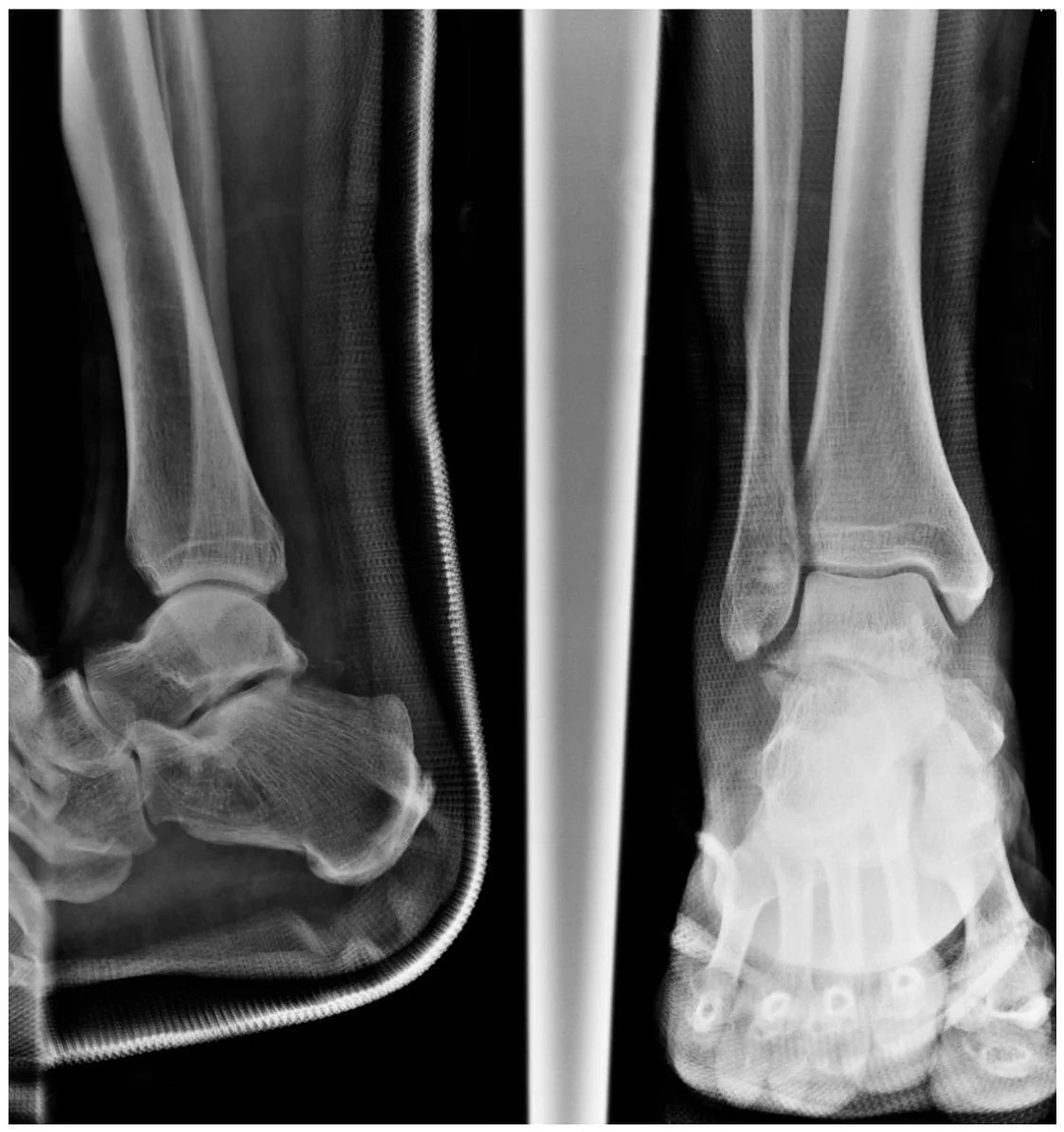
AP and lateral view upon admission.

**Figure 2:**
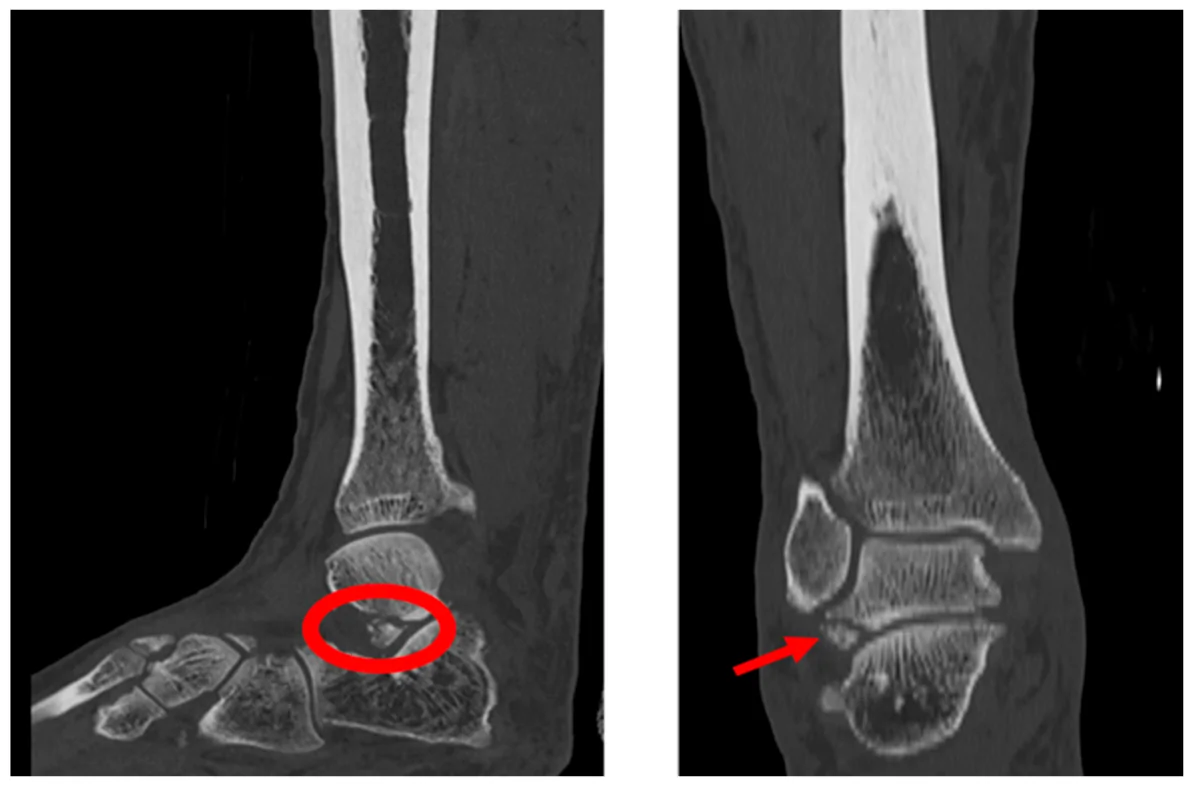
Computed tomography (CT) sagittal and coronal cross-section. The applied cast has been removed for imaging purposes. Red circle and arrow indicate the fracture.

**Figure 3:**
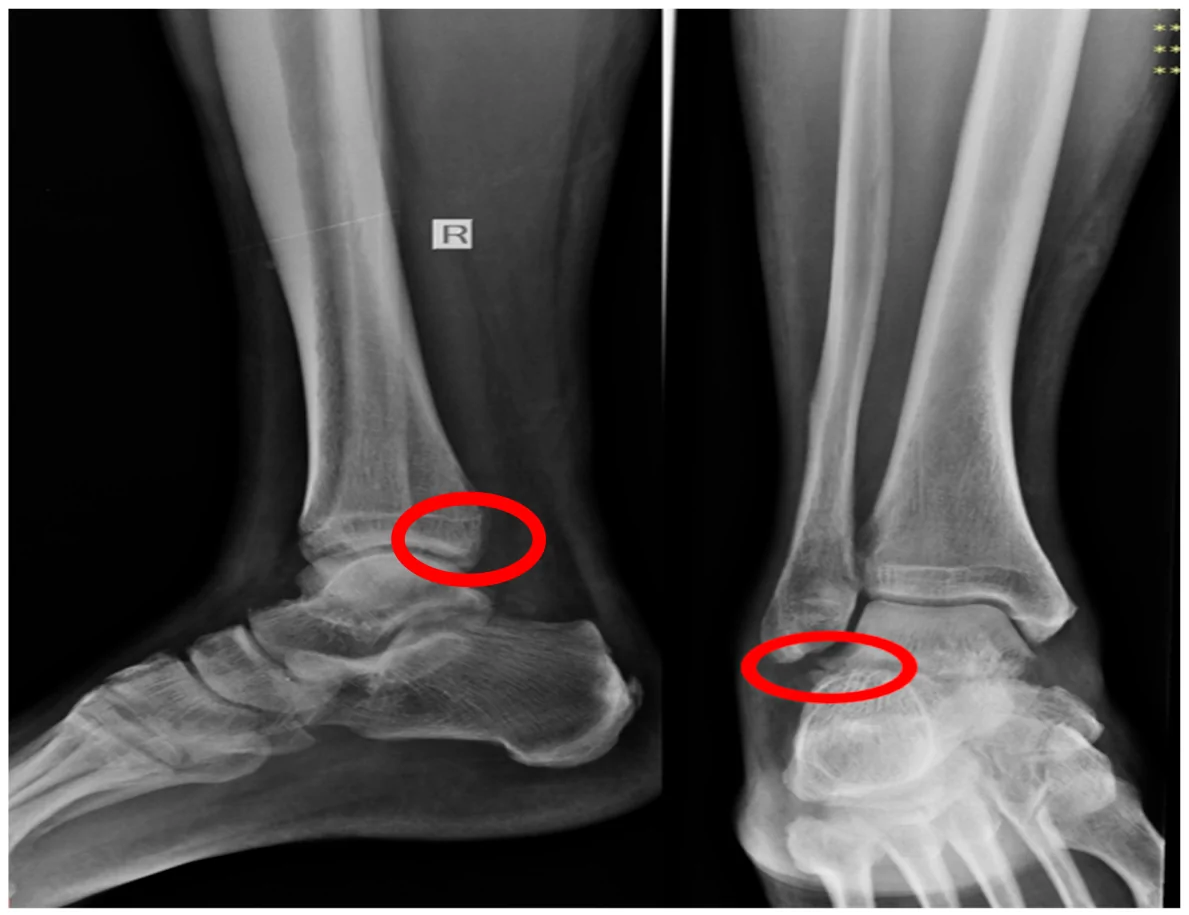
AP and lateral view of the Right (R) ankle at the 6-week follow-up point. Red circle outlines the fragment.

**Figure 4:**
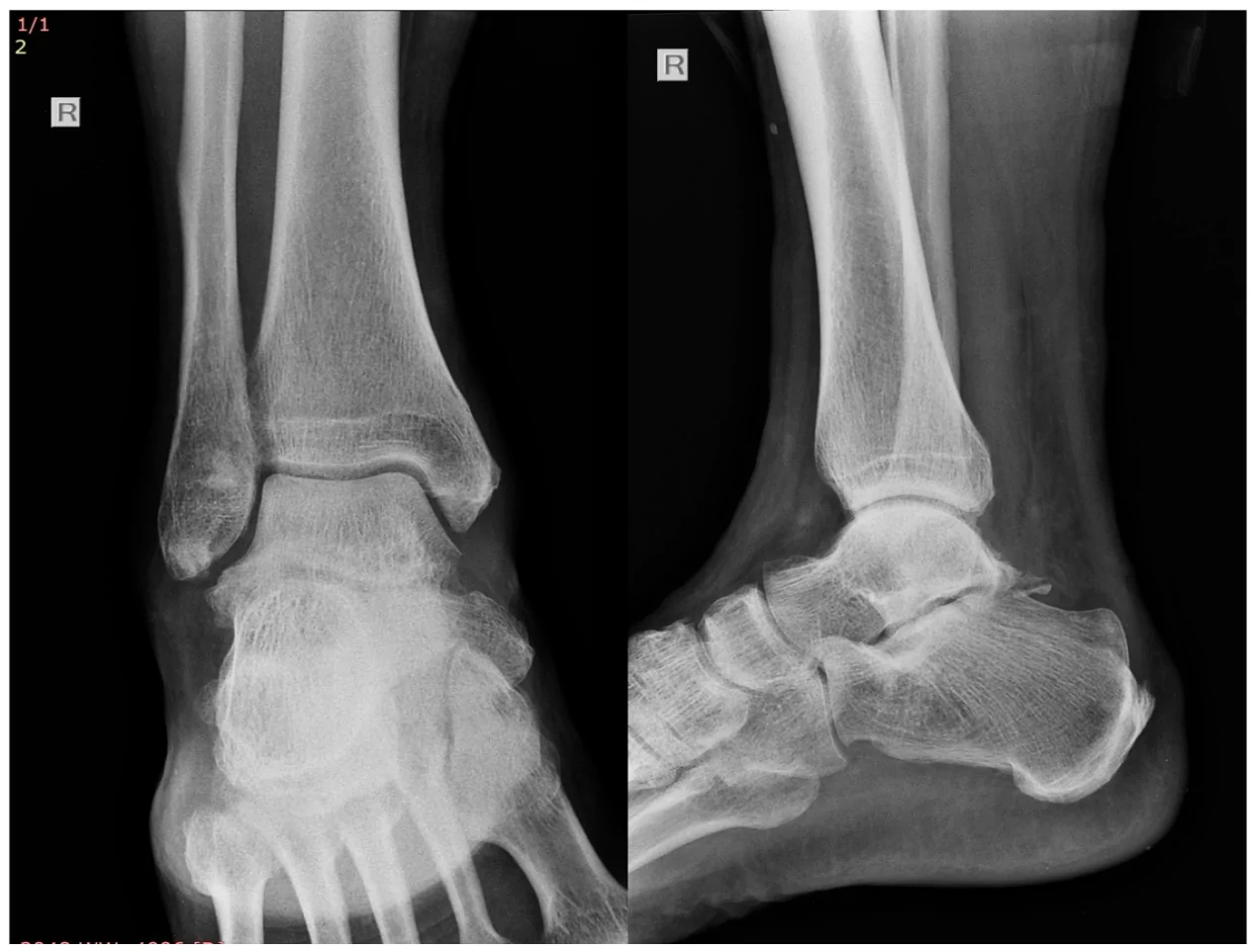
AP and lateral view of the Right (R) ankle in the annual follow-up visit. No fracture line is depicted.

**Figure 5:**
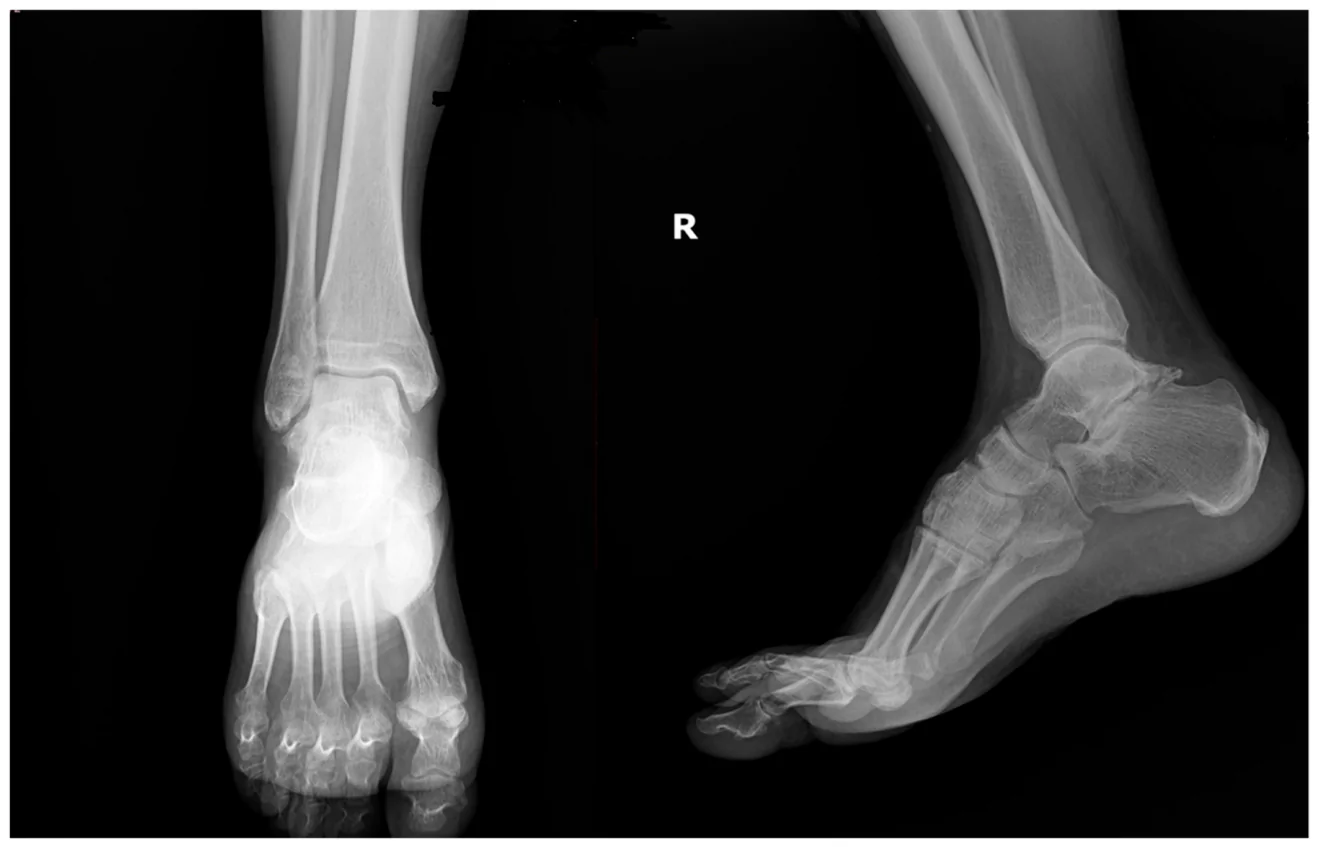
AP and lateral view of the Right (R) ankle at the 78-month follow-up point.

## Data Availability

The original contributions presented in this study are included in the article. Further inquiries can be directed to the corresponding author.
